# Transcriptional and Epigenetic Regulation of Monocyte and Macrophage Dysfunction by Chronic Alcohol Consumption

**DOI:** 10.3389/fimmu.2022.911951

**Published:** 2022-06-29

**Authors:** Delphine C. Malherbe, Ilhem Messaoudi

**Affiliations:** Department of Microbiology, Immunology and Molecular Genetics, College of Medicine, University of Kentucky, Lexington, KY, United States

**Keywords:** monocytes/macrophages, epigenetics, transcriptome (RNA-seq), inflammation, alcohol

## Abstract

Drinking alcohol, even in moderation, can affect the immune system. Studies have shown disproportionate effects of alcohol on circulating and tissue-resident myeloid cells (granulocytes, monocytes, macrophages, dendritic cells). These cells orchestrate the body’s first line of defense against microbial challenges as well as maintain tissue homeostasis and repair. Alcohol’s effects on these cells are dependent on exposure pattern, with acute drinking dampening but chronic drinking enhancing production of inflammatory mediators. Although chronic drinking is associated with heightened systemic inflammation, studies on tissue resident macrophage populations in several organs including the spleen, liver, brain, and lung have also shown compromised functional and metabolic capacities of these cells. Many of these effects are thought to be mediated by oxidative stress caused by alcohol and its metabolites which can directly impact the cellular epigenetic landscapes. In addition, since myeloid cells are relatively short-lived in circulation and are under constant repopulation from the bone marrow compartment, alcohol’s effects on bone marrow progenitors and hematopoiesis are important for understanding the impact of alcohol systemically on these myeloid populations. Alcohol-induced disruption of progenitor, circulating, and tissue resident myeloid populations contribute to the increased susceptibility of patients with alcohol use disorders to viral and bacterial infections. In this review, we provide an overview of the impact of chronic alcohol consumption on the function of monocytes and macrophages in host defense, tissue repair and inflammation. We then summarize our current understanding of the mechanisms underlying alcohol-induced disruption and examine changes in transcriptome and epigenome of monocytes and mcrophages. Overall, chronic alcohol consumption leads to hyper-inflammation concomitant with decreased microbial and wound healing responses by monocytes/macrophages due to a rewiring of the epigentic and transcriptional landscape. However, in advanced alcoholic liver disease, myeloid cells become immunosuppressed as a response to the surrounding hyper-inflammatory milieu. Therefore, the effect of chronic alcohol on the inflammatory response depends on disease state and the immune cell population.

## Introduction

### Burden of Alcohol Use Disorder

Per the National Institute on Alcohol Abuse and Alcoholism (NIAAA), alcohol use disorder (AUD) is “a medical condition characterized by an impaired ability to stop or control alcohol use despite adverse social, occupational, or health consequences” ranging from mild to severe. Lasting changes in the brain caused by alcohol misuse perpetuate AUD and make individuals vulnerable to relapse (1). AUD exerts a heavy burden on people’s health and the economy. According to the 2020 National Survey on Drug Use and Health (NSDUH), alcohol consumption is widespread in the USA with 138.5 million people 12 and older reporting drinking alcohol in 2020 and 50% of them drinking alcohol in the month preceding the survey. While a majority of individuals are considered moderate drinkers defined as consuming 2 alcoholic drinks/day or less for men and 1 drink/day for women, 6.4% of people reported engaging in heavy alcohol use in the month preceding the survey. Heavy alcohol use is defined as binge drinking on 5 or more days in the past 30 days with binge drinking defined as consuming ≥5 or more drinks in one sitting for men and ≥4 drinks in one sitting for women. Furthermore, it is estimated that 10.2% of American age 12 and older and 2.8% of adolescents ages 12–17 met the diagnosis of AUD in 2020 (2).

In the United States (U.S.), alcohol is the third-leading preventable cause of death, with an estimated annual death toll of 95,000 people (3). Furthermore, the economic cost of alcohol misuse was estimated to be $249.0 billion in 2010 (4). Adverse outcomes associated with heavy drinking are not limited to the U.S. In 2016, 5.3% of all global deaths, were attributable to alcohol drinking and alcohol abuse was the seventh-leading risk factor for premature death and disability worldwide. Of alcohol-related deaths in 2016, 21.3% were due to digestive diseases (liver cirrhosis, pancreatitis), 19% to cardiovascular diseases, and 12.9% to infectious diseases (including tuberculosis, pneumonia, and HIV/AIDS) (3). These data suggest dysregulated anti-microbial and inflammatory responses with chronic alcohol consumption.

### Importance of Myeloid Cells for Innate Immune Functions

During infection or inflammation, macrophages and monocytes are recruited rapidly to the affected tissues where they play a crucial role in the antimicrobial immune response *via* phagocytosis and secretion of immune mediators (cytokines, chemokines, and growth factors). Furthermore, monocytes and macrophages coordinate the recruitment of additional immune cell populations including lymphocytes (5, 6). Monocytes and macrophages play a critical role in tissue repair and wound healing by participating in the initial inflammatory response, clearance of injured tissue and invading pathogens, and the resolution phase (7).

Myeloid cell embryonic development occurs in three distinct waves in humans and in mice, involving first the yolk sac then the fetal liver, and finally the fetal bone marrow (5, 8). Yolk-sac derived macrophages seed multiple tissues where they persist for the life of the organism (9). In the adult bone marrow, monocytes develop from hematopoietic progenitor stem cells (HPSC) *via* progressively restricted lineage-committed progenitors (10, 11). Monopoeisis is well characterized in adult humans and mice, where two independent developmental pathways exist from common myeloid progenitors (CMP): *via* granulocyte-monocyte progenitors (GMP) to monocyte progenitors (MP) or *via* monocyte-DC progenitors (MDP) to common monocyte progenitors (cMoP). Mature monocytes develop from the MP and cMoP progenitor populations into the three well-defined subsets of classical, intermediate, and non-classical monocytes (8) based on their expression of CD14 and CD16. The three subsets have distinct yet sometimes overlapping functions. Classical monocytes (CD14^++^ CD16^-^) are involved in phagocytosis, innate sensing/immune responses, and migration. Intermediate monocytes (CD14^++^ CD16^+^) main functions are antigen presentation, cytokine secretion (TNF-α, IL-1β, and IL-6, following TLR stimulation), differentiation, and regulation of apoptosis. Non-classical monocytes (CD14^dim^ CD16^++^) are involved in anti-viral responses, complement and Fcγ-mediated phagocytosis, and adhesion (6).

While monocytes are relatively short-lived blood circulating phagocytes, macrophages are longer-lived cells that reside in tissues, including the brain, lung, liver, and intestine, where they perform a central role in antimicrobial responses as well as in tissue homeostasis and repair. Microglia are the tissue-resident macrophages in the brain where they comprise approximately 5-15% of cells (12). Microglia originate from yolk-sac progenitor cells and are maintained in adulthood by self-replenishment without the involvement of progenitor cells from the bone marrow (13, 14). Microglia have two main functions: immune defense of the central nervous system (CNS) *via* phagocytosis and mediator production as well as CNS maintenance *via* their control of neuronal proliferation (15). Lung macrophages are the most abundant immune cell population in the healthy lung and are composed of alveolar macrophages in the lumen and interstitial macrophages in the tissue (16, 17). Alveolar macrophages arise from fetal liver-derived monocytes and populate alveoli after birth. They are maintained by self-renewal without the contribution of bone marrow (16, 17). Alveolar macrophages participate in lung host defense and in gas exchange (16, 17). Kupffer cells are the liver tissue-resident macrophages and also are self-renewing populations originating from fetal-liver precursors (18). Other tissue-resident macrophage populations, including in the intestine (where they reside primarily in the lamina propria) (19) and the dermis (20), develop *via* fetal liver monocyte intermediates and are replenished in adulthood by monocyte-derived macrophages.

While chronic moderate alcohol consumption and acute alcohol exposure affect multiple cell populations and their functions, this review will focus on the impact of chronic heavy alcohol use on circulating monocytes and macrophages that reside within the brain, liver, intestine, and lung since a rich body of literature indicates that heavy alcohol consumption disproportionally impacts their phenotype and function (21, 22).

## Models Currently Available to Study the Impact of Chronic Alcohol Consumption on Monocytes and Macrophages

Findings from clinical studies are confounded by the presence of organ damage, smoking, use of recreational or illicit drugs, nutritional deficiencies, and by self-reported alcohol intake. To address these limitations, different experimental models have been developed to study the effects of alcohol on cell function in more defined settings. The main model systems are described below and listed in **Table 1**.

**Table 1:** Animal models of chronic alcohol exposure.

Model	Species	Alcohol administration	Characteristics
**Meadows-Cook (MC)**	Rodent	Ad libitum alcohol-drinking water	Mild steatosis
**Lieber-DeCarli (LD)**	Rodent	Ad libitum liquid diet	Mild inflammation Marked steatosis
**Tsukamoto-French**	Rodent	Enteral feeding	Mild inflammation Severe steatosis Fibrosis
**2^nd^ hit models**	Rodent	Ethanol exposure combined with LPS or high/fat diet	Fibrosis High mortality rate
**Voluntary drinking**	Nonhuman primate	Self-administration (22h/day)	Animals stratified based on blood ethanol concentration No overt liver damage after 12 months Fatty liver disease after 19 months
**Gastric infusion**	Nonhuman primate	Intragastric catheter (5h/4days or 0.5h/day for 3 months)	

### *In Vitro* Systems

To model ethanol consumption, primary and immortalized cells/cell lines are exposed *in vitro* to ethanol at different concentrations and for various durations (23, 24). Due to their ease of manipulation, several macrophage cell lines have been used to study the *in vitro* effect of alcohol exposure on cellular processes. Some notable examples include cell lines to model lung alveolar macrophages [mouse AM cell line MH-S cells (25), rat alveolar macrophage cell line NR8383 cells (26), and mouse macrophage cell line RAW264.7 (27)]; Kupffer cells [HepG2 human liver hepatocellular carcinoma (28), Huh7 human hepatoma cells (29), rat Kupffer cell line 1 (RKC1) (30), human macrophage cell lines MonoMac6 (31), and mouse J774.1 cell line (32)]; microglia [mouse BV2 cell line (33)], rat cell line Microglia-SV40 (34)). In addition, THP-1 human cells differentiated into macrophages have been extensively used (35). Primary cells, including alveolar macrophages, hepatocytes and peripheral blood mononuclear cells (PBMCs) obtained from control and alcohol-exposed animals and humans have also been used (24).

However, transformed cell lines respond differently to immunological challenges compared to primary cells (36) and *in vitro* exposure to ethanol does not accurately recapitulate the complexity of *in vivo* alcohol consumption such as the generation of ethanol metabolites (37). To address some of these challenges, organoid models have been developed, offering new opportunities to explore the impact of chronic ethanol exposure using three-dimensional (3D) *in vitro* structures that better replicate the cellular complexity as well as the morphological and functional features of *in vivo* tissues compared to cells grown in a monolayer (38). Organoid models for organs heavily impacted by alcohol consumption (including liver, brain, intestine, colon, lung) are now available (39–45). A recent liver organoid model replicates alcoholic liver disease (ALD)-associated pathophysiologic changes upon ethanol treatment, including oxidative stress response, steatosis, inflammatory mediators release, and fibrosis in hepatocytes (39). Brain organoids (40) were recently used to assess the effect of alcohol binge drinking on neurons and astrocytes (46). Alcohol-mediated neurotoxicity was unveiled at the structural, functional, and transcriptional levels, revealing that alcohol-induced dose-dependent apoptosis was more severe in neurons than in astrocytes (46). Gut organoids have also been developed including a colon organoid based on a normal colon epithelium (41, 42). Jejunum and colon organoids showed that alcohol’s effects mainly targeted colon cells leading to gut leakiness (43) in line with findings showing that ALD patients with the worse intestinal dysbiosis exhibit the greatest gut leakiness (47). While alcohol’s effect on epithelial and endothelial cells was assessed in these various organoids, the role of macrophages in alcohol-mediated disease was not investigated even though they are a prominent cell subset in the colon, brain, and liver (18).

### Rodent Models

Mice and rats are the animal models most commonly used to study the impact of chronic alcohol consumption on several organ systems. Depending on the model, ethanol is administered *via* different modalities (liquid diet, ethanol in water, gavage, or injection) in combination with dietary, chemical, or genetic manipulations (48, 49). Two prominent mouse models are the Lieber–DeCarli liquid diet (LD) and the Meadows-Cook diet (MC) of *ad libitum* ethanol feeding (50, 51). The LD model, designed to enhance the alcoholic liver injury phenotype in mice (50, 52), is based on an isocaloric liquid diet with alcohol concentration usually increased from 0% to 3.395% w/v for a period of 25 days to 8 weeks, with a 4-week duration most commonly used. In contrast, in the MC diet where alcohol is delivered in the water, exposure to alcohol consists of a 2-week ramping up phase from 0% to 20% ethanol followed by a maintenance phase of 4–16 weeks with a 12-week duration most commonly used (51). Some cellular immunological changes are model-dependent and limited common shared alcohol-induced transcriptional abnormalities are observed between LD and MC models (53). In addition, these mouse models of chronic alcohol consumption are characterized by minimal liver inflammation (52). Thus, both MC and LD mouse models are models of early alcoholic liver injury (steatosis) and not models of more advanced liver diseases such as fibrosis, cirrhosis, or alcoholic hepatitis. Moreover, the aversion to self-administration of ethanol in some mouse strains (BALB/c resistance vs C57BL/6) as well rodents’ higher alcohol catabolism rate compared to humans, make the analysis of organ damage challenging in a self-administration setting (54, 55).

To address these limitations, models delivering ethanol *via* oral gavage or intragastric injection were established, including the Tsukamoto-French enteral feeding model that results in moderate liver inflammation and steatosis (55). To model more advanced ALD while using the oral delivery route and drinking patterns closer to those observed in humans, second-hit models were developed that combine ethanol exposure with a secondary stimulus including genetic knockout/overexpression, LPS stimulation, or high-fat diet (49). Furthermore, additional mouse models were established to study specific aspects of AUD such as binge drinking with the drinking in the dark model (56); a voluntary escalation in consumption with the chronic two-bottle choice drinking (57); and development of dependence with the chronic intermittent vapor model (58). In addition to mouse models, rat models of ethanol drinking have also been developed, including the alcohol-preferring (P) and the high-alcohol-drink (HAD) rat models which prefer an alcohol solution containing 10% ethanol over water (59).

### Nonhuman Primate Models

Nonhuman primate (NHP) models are the gold standard to study human diseases due to their genetic proximity to humans; therefore different NHP models of ethanol delivery were developed to mimic human alcohol consumption. Models of oral ethanol self-administration include some with stress triggers including shock avoidance, food and water deprivation, or social stressors (60–62) as well as those with positive reinforcement triggers (63). In one of the models of voluntary ethanol self-administration using a positive reinforcement trigger, rhesus macaques are first induced to freely consume increasing daily doses of ethanol, ranging from 0.5 g/kg/day to 1.5 g/kg/day over a 3-month phase followed by “open access” to a 4% w/v ethanol solution for 22 h/day. This approach establishes consistent ethanol self-administration, and thus represents a true model of alcohol addiction in rhesus and cynomolgus macaques (64, 65) with animals being categorized as low drinkers (average ethanol intake less than 2 g/kg), binge drinkers (average intake of 2.4 g/kg), heavy drinkers (average intake of 2.8 g/kg) or very heavy drinkers (intake higher than 3 g/kg) (66). While this experimental model does not induce any overt liver damage over a 12-month period, signs of fatty liver disease were observed in cynomolgus macaques exposed to an open access period of 6 months followed by 12 months of abstinence that was followed by another open access period of up to 19 months (67). This rhesus macaque model was used to define the impact of chronic drinking on circulating and tissue-resident immune cells showing that the circulating innate immune cells bear the largest burden of chronic heavy drinking (21, 22, 68–73). However due to the inherent variation in alcohol consumed in the voluntary drinking models, a model of gastric infusion of alcohol was developed in order to deliver a consistent alcohol dose in treated rhesus macaques, where animals are infused with 30% ethanol in water (w/v) *via* an intragastric catheter either 4 days per week or 7 days per week during 3 months resulting in blood alcohol levels of 2.3 g/kg (74–76). This model has been used to study the impact of alcohol consumption on simian immunodeficiency virus (SIV) infection (74–76).

## Impact of Alcohol Consumption on Susceptibility to Infection and Wound Healing

### Infection

Chronic alcohol consumption weakens antimicrobial responses by impairing the immune system’s responses and by affecting the integrity of the mucosal barrier resulting in increased susceptibility to bacterial and viral infections (77–80). In particular, chronic alcohol consumption is a predisposing factor for severe respiratory infections, such as bacterial pneumonia, respiratory syncytial virus and SARS-CoV-2 infections (81–86). Several mechanisms contribute to increased disease severity, including alcohol-induced impairment of the innate immune response, chronic oxidative stress, and alcohol-induced organ damage due to excessive inflammation (82). In addition, chronic alcohol consumption affects lung physiology by desensitizing lung airway cilia and compromising the mucociliary escalator, and leading to impaired clearance of invading pathogens (82).

Community-acquired pneumonia is a leading cause of death in the USA and patients who suffer from AUD experience more severe pneumonia than non-AUD patients (77, 81, 83, 87). Streptococcus pneumoniae, usually a commensal bacterium in the upper respiratory tract, is the prevalent infectious agent of community-acquired pneumonia (81) but alcohol abuse is also associated with an increased incidence of pneumonia from gastric aspiration of Klebsiella pneumoniae (82, 83). Moreover, alcohol abuse is a risk factor for active tuberculosis (TB) with a three-fold risk increase of active TB associated with heavy drinking (80, 88) as well as reactivation of latent tuberculosis (80, 88, 89). AUD patients are also at a higher risk of viral infections, notably respiratory syncytial virus (RSV) (84) and SARS-CoV-2 (85, 86). Increased severity of COVID-19 is believed to be additionally mediated by increased prevalence of cardiovascular diseases and chronic respiratory diseases with alcohol abuse (85, 86). Finally, chronic alcohol consumption is an independent risk factor to develop acute respiratory distress syndrome (ARDS) (90). ARDS is characterized by endothelial and alveolar epithelial barrier dysfunction, severe inflammation, and pulmonary surfactant dysfunction leading to impaired gas exchange in the lung and insufficient oxygen levels in the blood and tissues resulting in organ failure (82).

Infectious diseases affecting the liver are also more prevalent in AUD patients as alcohol metabolism takes place in the liver (91). Specifically, the prevalence of hepatitis C virus (HCV) infection is up to 30-fold higher in AUD patients compared with the general population (92), with a 4.6% to 55.5% rate in AUD patients (93). Moreover, chronic alcohol has been reported to enhance the replication of hepatitis B virus and possibly HCV (93). The increased susceptibility is also mediated by an alcohol-induced increase in oxidative stress and impaired antiviral immune responses in the liver (91, 94). In addition to respiratory and liver diseases, alcohol abuse is also positively associated with HIV incidence and progression towards AIDS in part due to antiretroviral therapy failure (95). Moreover, alcohol increases the risk of multiple AIDS comorbidities including hepatic fibrosis, hepatic cirrhosis, neurocognitive impairment, and AIDS-related dementia (96).

### Wound Healing

Wound healing is a dynamic and tightly coordinated process defined by three interconnected phases: inflammation, proliferation, and remodeling. Studies have revealed the critical role played by myeloid cells at several steps of this process. Inflammation is the first phase where neutrophils and macrophages are recruited to the injury site to protect it from infection by phagocytosing invading pathogens and by producing reactive oxygen species (ROS) and pro-inflammatory cytokines and chemokines. These mediators are produced both by local tissue-resident macrophages and by circulating monocytes being recruited from bone marrow to differentiate into macrophages at the site of injury (7). After this initial pro-inflammatory response, macrophages switch to an anti-inflammatory phenotype and participate in wound resolution *via* their contribution to angiogenesis and formation of fibrous tissue (97). During the proliferative second phase, cell migration and proliferation are upregulated to repopulate the injured tissue with fibroblasts, endothelial and epithelial cells. Tissue reorganization takes place last during the remodeling phase so that the injured tissue can regain its functionality (98).

Alcohol abuse impairs wound healing by affecting each step of the repair process (97–99) resulting in AUD patients recovering poorly from surgeries and traumas. AUD patients with a burn injury require longer hospitalizations with more aggressive antibiotic treatments (100), exhibit higher rates of secondary bacterial infections, and are more likely to die compared to non-AUD burn patients (101, 102) even from smaller burn injuries (100). In case of other types of trauma, findings are conflicting with some studies showing that acute rather than chronic alcohol consumption accounts for septic complications after abdominal penetrating trauma while others found that chronic but not acute alcohol consumption influences the outcome from trauma (103). Alcohol consumption is associated with an increased risk for *Staphylococcus aureus* infection of the skin, further compromising the response to wound healing (82). Chronic alcohol exposure may impair wound healing by preventing macrophages from switching to a regulatory phenotype, a critical step to enable the subsequent resolution phase of the healing process (104). Indeed, alcohol exposure induces alterations in transcription and chromatin accessibility in alveolar macrophages indicative of decreased capability for tissue repair (105). In addition, alcohol-exposed macrophages have a reduced phagocytic capacity (26, 105) and this could further impair the wound healing process by reducing the clearance of invading pathogens (104).

## Impact of Chronic Ethanol Consumption on the Functional and Transcriptional Landscape of Monocytes and Macrophages

### Altered Inflammatory Response

**Figure 1:**
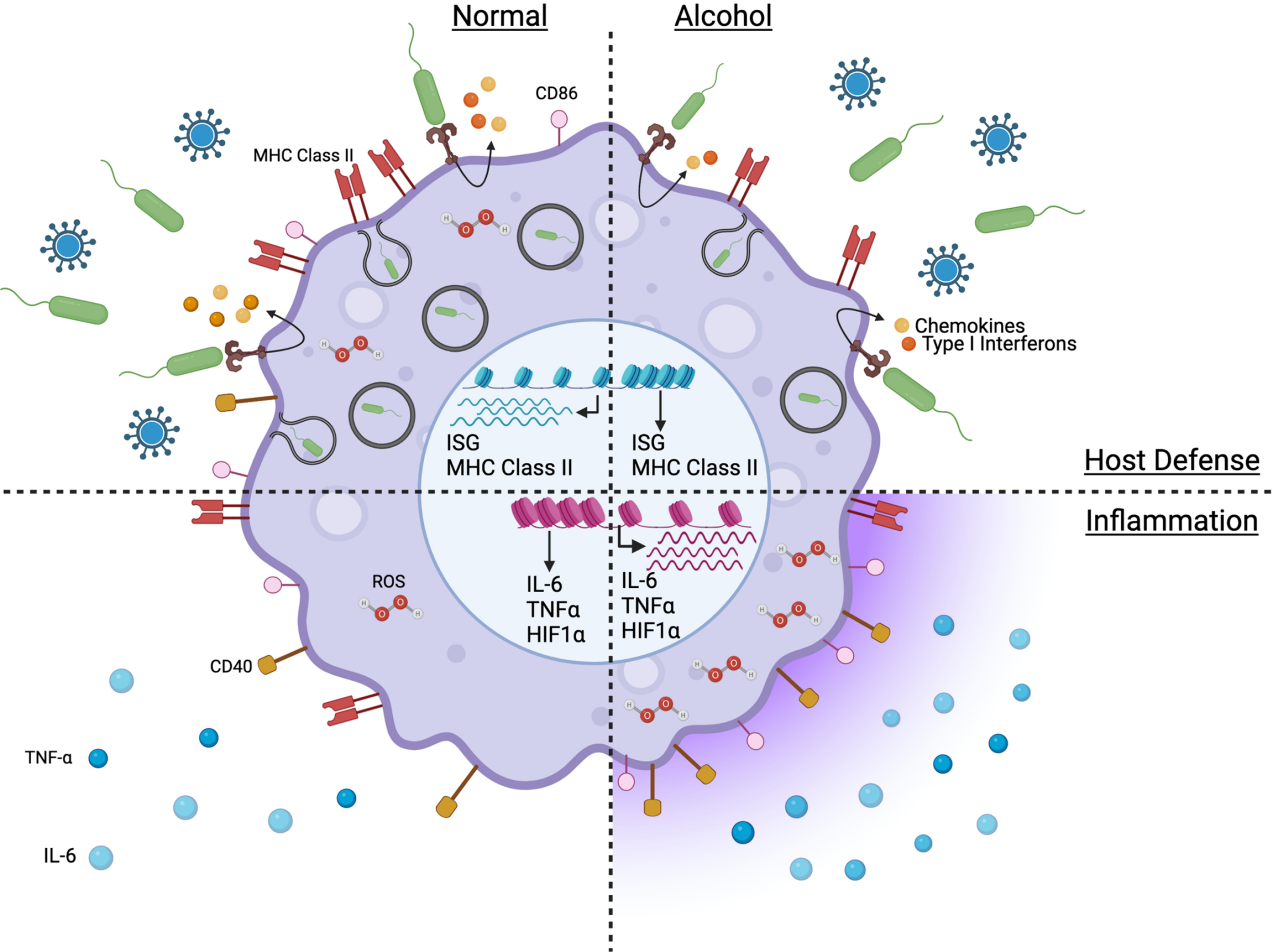
Model capturing the impact of chronic alcohol consumption on the phenotype and function of monocytes and macrophages in absence of severe alcoholic liver disease. Figure created withBioRender.com.

The effect of chronic alcohol on the inflammatory response depends on the disease phase (early vs late) and the cell populations affected (tissue macrophages vs circulating monocytes). A pro-inflammatory cytokine profile (**Figure 1**) is observed in AUD patients, with elevated plasma levels of TNF-α, IL-1β, and IL-6 (106). Similarly, chronic alcohol results in a systemic pro-inflammatory response in ethanol-fed mice with increased circulating protein levels of TNF-α, IL-6 and MCP-1 (107). This heightened inflammatory state has been associated with multi-organ damage affecting the liver, intestine, lung, and brain (108–111). Prolonged *in vitro* exposure to ethanol upregulates TNF-α gene expression in human monocytes (112). Hyper-inflammation and heightened cell activation have also been observed in HIV patients suffering from AUD with increased plasma levels of soluble CD40 and TGF-β as well as upregulation of CD16, CD163 and TLR4 expression on monocytes (113). In addition, chronic alcohol consumption increases human monocyte sensitivity to LPS as indicated by elevated secretion of pro-inflammatory mediator TNF-α in part *via* downregulation of IRAK-M and enhanced activation of NF-κB and ERK kinases (23, 114).

Inflammatory responses are imbalanced in alcoholic hepatitis (AH) and acute-on-chronic liver failure (ACLF), two severe conditions with substantial mortality and morbidity observed in patients with significant AUD. While AH was initially associated with a proinflammatory phenotype due to higher circulating cytokines levels (115–117), recent studies revealed that AH leads to monocyte immuneparesis characterized by a downregulation of HLA-DR, TNF-α, Il-1β and IL-6; upregulation of regulatory IL-10, PPARγ and MERTK that were accompanied by a decrease in non-classical monocytes and increase in intermediate monocytes (118). Indeed, at the transcription level, monocytes from patients with severe AH display a phenotype characterized by immunosuppressive features including reduced expression of genes related to immune pathways (innate immune responses, cytokines, response to IFNs, antigenic presentation, phagocytosis) (118). Similarly, immuneparesis was also observed in ACLF monocytes with downregulation of HLA-DR and CD86; and upregulation of regulatory IL-10 and MERTK while classical monocyte populations decreased and intermediate monocytes increased (119, 120). The seemingly contradiction of a systemic pro-inflammatory milieu and immunosuppressed cells could be due to an environment with heightened release of soluble proinflammatory mediators by alcohol-injured tissues leading to expansion of mononuclear CD14^+^HLA-DR^-^ myeloid-derived suppressor cells resulting in immunosuppression of monocytes in an attempt to reduce the inflammation (121–123). This immuneparesis extended to peritoneal and mesenteric lymph node macrophages and Kupffer cells in ACLF patients (119).

Furthermore, single-cell transcriptome analyses performed at rest revealed an unsuspected level of heterogeneity in classical monocyte subsets of AH patients with three distinct classical monocyte clusters (124). These three classical clusters differentially expressed markers including CD14, MHC class II, TLR8, and CD86 (124). Interestingly, this high level of heterogeneity in classical monocytes was also observed in alcohol-fed NHPs with the identification of seven classical monocyte clusters by single cell RNA sequencing analysis (68). Two of these clusters were exclusively composed of cells from chronic heavy drinking macaques: *HIF1A* ^hi^ and *SOD2* ^hi^. Marker genes of these clusters enriched to gene ontology terms related to myeloid cell differentiation, cytokine signaling, and response to alcohol. Trajectory analysis showed that chronic heavy drinking was associated with a specific lineage that culminated in the *HIF1A* ^hi^ cluster, indicating that heavy drinking dysregulates monocyte differentiation states thereby affecting monocyte response to inflammation (68). In addition, chronic heavy drinking was associated with downregulated expression of MHC class II genes but upregulated expression of IFN-inducible genes and activation of NF-κB signaling pathway in classical monocytes (68).

In alcohol-fed animal models, chronic drinking also results in pro-inflammatory responses by Kupffer cells, microglia, splenic and lung alveolar macrophages (22, 82, 107, 125–129). Ethanol-fed mice upregulate the expression of *Tlr1*, *2*, *4*, *6-9* genes leading to an increase in *Tnf* gene expression (130). Furthermore, alcohol-fed mice deficient in TLR4, CD14, TNF-α or LPS-binding proteins (LBP) have reduced liver inflammation and do not develop liver injury (131–133). Anti-TNF-α antibody treatment prevented liver inflammation in alcohol-fed rats (134). Chronic heavy drinking in rhesus macaques is associated with higher gene expression of pattern recognition receptors (*TLR4, FPR3, TLR2)*, chemokine receptors (*CCR1, CCR5*), differentiation and activation markers (*CD163*) in splenic macrophages (22). This effect is mediated by alcohol-induced enhanced chromatin accessibility of stress-responsive transcription factors that regulate genes involved in macrophage phagocytosis (*GATA-2*), activation (*CEBPA*), hypoxia (*HIF-1α*), polarization (*GATA-3, NFAT5*) and pro-inflammatory cytokine production (*GATA-2, NRF-1, NFAT5*) (22). In addition, chronic alcohol enhances chromatin accessibility of promoters that regulate genes involved in pro-inflammatory responses, including *TLR4* and *CCL2* genes (22). Chronic alcohol exposure also increases access to transcription factors that regulate expression of inflammatory genes in alveolar macrophages, including AP-1, IRF8, and NF-κB. In the lung, chronic alcohol also upregulates genes involved in granulocyte activation and degranulation (*CTSG, SNAP25, MYO3A*); and downregulates genes involved in anti-inflammation (*CLEC1B*) (105). At the single cell level, chronic alcohol increased the expression of genes involved in oxidative stress (*LCN2*), *HIF-1α*, and cytokine signaling (105).

Alcohol consumption also elicits functional and transcriptial changes in the microglia, with high expression of *TLR2* gene in alcoholic human brains (135). Moreover, alcohol treatment of mice upregulates expression of *Tlr3* in the cortex where microglia are found (136) and chronic intermittent ethanol vapor exposure leads to upregulation of type I interferon-stimulated genes in the prefrontal cortex of mice (137). Additionally, gene ontology analysis identified “toll-like receptor signaling pathway” and “activation of innate immune response” as the main processes in microglia affected by alcohol exposure (137, 138). Chronic alcohol induces transcription of pro-inflammatory IL-6, IL-1β, TNF-α, and MCP-1 in mouse brain tissue, cortex, and isolated microglial cells *via* NF-κB activation (136, 138). In addition, levels of anti-inflammatory IL-10 were decreased in a mouse model of chronic alcohol-induced neuroinflammation (139).

LPS stimulation of alcohol-exposed monocytes and tissue macrophages further enhances the alcohol-induced hyper-inflammatory response. Upon LPS stimulation, monocytes of alcohol-fed NHPs upregulate genes associated with myeloid inflammatory pathways (*TNFSF21*, *TLR2*). These transcriptional pro-inflammatory changes were confirmed at the protein level with the increased production of pro-inflammatory mediators TNF-α, IL-6, IL-15, and CCL4 in LPS-stimulated monocytes (68). In splenic macrophages isolated from male macaques classified as chronic heavy drinkers, LPS stimulation induced greater production of TNF-α, MIP-1α, MIP-1β, IL-1β, IL-6, IL-8, and GM-CSF (22). From a transcriptional standpoint, LPS-responsive transcription factors NF-KB, p65, STAT1, SMAD1, and HIF-1 were upregulated along with genes associated with “innate immune response”, “TGF-β signaling” and “response to LPS” including *CD80, NFKB1, JUN, IL6ST, IFNG, TNFRSF8* and *TNFRSF10B* and *SOCS1* (22).

Alcohol-induced altered inflammatory state following LPS stimulation is not limited to circulating monocytes or macrophages from lymphoid tissues. Chronic heavy ethanol consumption for 12 months leads to a hyper-inflammatory response to LPS stimulation by NHP alveolar macrophages with increased production of IL-6, TNF-α, CXCL8, CXCL10, CCL2, and CCL4 (105). Similarly, chronic alcohol consumption increased protein levels of IFN-α and IFN-β in bronchoalveolar lavage (BAL) of mice. In addition, alveolar macrophages from alcohol-fed mice infected with RSV secrete higher levels of MCP-1 and TNF-α (84). In mice, chronic alcohol also leads to increased expression of pro-inflammatory mediators IL-6, TNF-α, CXCL-1 and MMP-9 by liver-resident Kupffer cells (107). Alcohol-induced liver inflammation in mice and rats is mediated by Kupffer cells, as suggested by upregulation of cell surface expression of CD14 and increased production of pro-inflammatory mediators TNFα, MCP-1, and reactive oxygen species (ROS) (140–145). Both alcohol-induced ROS and increased Kupffer-cell sensitization to endotoxin further exacerbate proinflammatory responses and are major drivers of ALD (91, 144). Intestinal macrophages from alcohol-exposed mice express higher levels of pro-inflammatory genes both at rest and following LPS-induced systemic inflammation, including *Lcn2*, *Tnf*, *Il1b* and *Csf1* (146). *Lcn2* gene encodes Lipocalin-2 which plays a pro-inflammatory role in metabolic diseases (147). *Csf1* plays a role in the maintenance of intestinal macrophage populations (148), thus suggesting that alcohol-induced inflammation may increase the number of resident intestinal macrophages (147). In addition, LPS stimulation of alcohol-fed mice increases the expression of pro-inflammatory chemokine gene *Cxcl1* (neutrophil recruitment) and cytokine signaling gene *Il1a* and its receptor *Il1r* in the ileum (146).

One of the potential drivers of the heightened inflammatory response following chronic alcohol exposure is microbial translocation and leakage of endotoxin in the portal circulation driven by impaired intestinal barrier function and increased gut permeability (149, 150). Additionally, alcohol abuse leads to bacterial overgrowth (151) and altered microbiome composition in the gut of alcoholic patients and alcohol-fed mice (152–154). Fecal transplants from alcohol-fed mice into germ-free mice induced intestinal inflammation, leaky gut, and liver injury (155), thus highlighting the role of dysbiosis in alcohol-induced inflammation. These effects are mediated by bacterial toxins including cytolysin as demonstrated by inhibition of alcohol-induced liver injury in humanized mice by bacteriophage treatment targeting cytolysin-producing *E.faecalis* (156). In addition, plasma endotoxin levels correlated with liver inflammation in alcohol-fed rats (157). While endotoxin leakage has been observed in some mouse models of chronic alcohol consumption (152, 158, 159), there is no consistent increase in endotoxin levels in plasma or in antibody-bound endotoxin levels in NHPs after 12 month of chronic ethanol consumption suggesting that a longer duration may be necessary to detect significant dysbiosis and translocation of microbial products in this model (68, 153).

### Defects in Phagocytosis and Antiviral Responses

Defects in anti-microbial functions of monocytes and macrophages (**Figure 1**) have been reported to play a critical role in the observed increased vulnerability to infectious diseases due to chronic drinking resulting in reduced phagocytic activity and pathogen-fighting function in Kupffer cells, microglia, alveolar and splenic macrophages (22, 107, 125–128). Under homeostatic conditions, chronic alcohol leads to a transcriptional downregulation of genes associated with immune and anti-viral responses (MHC class II, antigen processing and presentation, and IFNγ signaling pathways) in non-classical monocytes of alcohol-fed NHPs (68). In contrast to the hyper-inflammatory response to LPS generated by monocytes/macrophages from heavy drinking humans or animals, chronic alcohol exposure leads to a dampened production of immune mediators by macaque monocytes stimulated with *E.coli* bacteria, including reduced production of IL-1β, IL-5, IL-6, IL-15, CCL4 and CXCL11 (68). In addition, genes associated with adaptive immune activation (*IL21R, CD40, MHC class II*) are downregulated in LPS-stimulated monocytes from heavy drinking NHPs (68). Similarly, in splenic macrophages, LPS stimulation leads to downregulation of genes associated with “type I interferon signaling”, “defense response to virus” (22). Furthermore, monocytes from AH patients upregulate SOCS1 (160), a negative regulator of the JAK/STAT pathway (161), leading to impaired IFNγ signaling and impaired intracellular killing of phagocytosed bacteria in a subset of patients and is associated with poor survival outcomes (160). Chronic alcohol also led to reduced immune response to vaccination in NHPs and downregulation of several innate immune genes predicted to be highly expressed by myeloid cells including genes involved in antigen presentation (MHC class II genes), defense responses (type I IFN) as well as microbial sensors (*CD14, TLR4, TLR5*) (71).

In addition to impairing antimicrobial functions of monocytes and macrophages of lymphoid organs, chronic alcohol also alters the function of tissue-resident macrophage populations, notably alveolar macrophages, Kupffer cells, and microglia, resulting in increased disease severity (94). Patients with AUD are more susceptible to respiratory infections (81–86) and alcohol-fed mice infected with Mycobacterium tuberculosis have a higher lung bacterial load and a dampened IFN-γ production (162). In addition, chronic alcohol exposure impairs phagocytosis of bacteria by alveolar macrophages, such as *E.coli* by rat macrophages and *S.aureus* by NHP macrophages (26, 105). Chronic alcohol consumption also leads to blunted induction of interferon-stimulated genes (ISGs) by alveolar macrophages in response to RSV in NHPs suggesting disrupted anti-viral responses (105). Similarly, in a mouse model of chronic ethanol consumption, RSV infection resulted in increased viral loads in the lung and reduced IFNγ protein levels in bronchoalveolar lavage (84). Influenza infection of alcohol-fed mice leads to increased lung viral loads and disease severity (163). In addition, alcohol-fed mice infected with *Mycobacterium avium* have higher bacterial burden in the liver, spleen, and blood (164); and chronic alcohol has been shown to reduce *E.coli* phagocytosis by rat Kupffer cells and microglia (165, 166). Therefore, chronic alcohol dampens microbe phagocytosis by multiple tissue-resident macrophage populations, leading to defects in antimicrobial responses in rodents and NHPs exposed to chronic alcohol.

### Alcohol and Increased Oxidative Stress Response

ROS are important mediators in cell signaling and apoptosis (167) and play a critical role in the pathogenesis of various diseases including respiratory infections. ROS are produced by NADPH oxidases (Noxes) (167, 168). Chronic drinking upregulates Nox expression resulting in elevated ROS production leading to heightened oxidative stress in Kupffer cells, microglia and brain cortex, alveolar and splenic macrophages (22, 107, 125–128, 136, 169). One proposed mechanism is *via* alcohol-mediated downregulation of PPARγ. This downregulation of PPARγ in alveolar macrophages following chronic alcohol exposure results in upregulation of Nox and an increase in oxidative stress response leading to heightened intracellular ROS levels (105). In addition, ROS increases the expression of transcription factors HIF-1α and HIF-2α which have important roles in the response to hypoxia in several tissues including mouse liver and rat brain cortex (170, 171). Furthermore, chronic alcohol elicits upregulation of genes enriched in the HIF-1α signaling pathway in alveolar and splenic macrophages as well as monocytes of ethanol-fed NHPs (22, 68, 105). Under homeostatic conditions, chronic alcohol also leads to increased differentiation of classical monocytes expressing high levels of HIF-1α (68). Alcohol’s effect on HIF-1α expression in the intestine is more complex with an increased HIF-1α expression in mice exposed to chronic alcohol for 28 days but a decreased expression when alcohol exposure is prolonged to 42 days (159). Therefore, overall chronic alcohol increases tissue hypoxia but its effect can be organ- and exposure-dependent.

## Mechanisms Underlying Alcohol-Induced Disruption of Monocyte and Macrophage Function

The functional effects of alcohol on myeloid cells are dictated by changes at the epigenome level that affect gene transcription (172). Regulation of gene expression can be mediated by epigenetic changes resulting in DNA modification that does not alter the genome sequence. The main epigenetic modifications are provided by noncoding RNAs, DNA methylation, and modifications of histones. MicroRNA molecules (miRNA) are short (19-25 nt), highly conserved, single-stranded non-coding RNAs that modulate target proteins by regulating mRNA expression through decreased transcription or by post-transcriptionally induced mRNA decay (173). In addition to having an autocrine effect, miRNAs can have a paracrine effect *via* exosomal delivery (174). DNA methylation is an epigenetic mark that targets cytosine residues of cytosine-guanine dinucleotide repeats and is regulated by DNA methyltransferases (DNMT) and ten-eleven translocation (TET) enzymes. Hyper-methylation in promoter gene regions leads to transcriptional repression and down-regulation of protein production (175). Histone proteins play key structural and regulatory roles by enabling the formation of nucleosomes. Histone modifications (methylation, phosphorylation, acetylation, and deacetylation as well as ubiquitination) impact chromatin packing thereby modulating gene expression (176). Histone acetyltransferases (HAT) increase chromatin accessibility and promote transcription, whereas histone deacetylases (HDAC) decrease chromatin accessibility and inhibit transcription. In contrast, the result of histone methylation is more complex and depends on several factors including the degree of methylation, the histone and lysine residue targeted, the level of chromatin condensation as well as the function of the genome region targeted (i.e. promoter, enhancer). H3K4, H3K9, and H3K27 are some of the most prominent lysine residues targeted. Studies have reported that alcohol can interfere with the fundamental processes of epigenetic regulation in several cell types including monocytes and macrophages in a dose-, duration-, and time-dependent manner (176).

### Role of MicroRNAs in Alcohol-Mediated Disruption of Monocyte and Macrophage Function

miRNAs play a critical role in regulating the function of macrophages and inflammatory pathways in alcoholic steatohepatitis (174). Indeed, several miRNAs modulate the alcohol-induced hyper-inflammatory response by inhibiting negative regulators of TLR signaling pathways (177) in multiple tissue-resident macrophages populations including Kupffer cells, microglia and alveolar macrophages. Alcohol exposure upregulates miR-155 and miR-132 in murine Kupffer cells (178–180) and microglia (181). miR-155 inhibits negative regulators of the TLR4 pathway, including IRAK-M, SHIP1, and PU.1, resulting in increased sensitization to LPS and increased production of pro-inflammatory TNF-α (179) in liver and TNF-α and MCP1 (CCL2) by microglia (181). In addition, miR-155 downregulates STAT3 and SOCS1 in murine Kupffer cells resulting in upregulation of pro-inflammatory cytokines TNF-α and IL-1β and downregulation of anti-inflammatory cytokine IL-10 (161, 179, 182). On the other hand, miR-132 regulates expression of TGF-β, IL-1β, and MCP-1 in Kupffer cells as well as TNF-α and MCP1 in the cerebellum (180, 181). Increased levels of TGF-β are key for the development of fibrogenesis in the liver, while increased levels of inflammatory mediators (IL-1β, TNF-α, MCP-1) contribute to increased hepatic inflammation and development of steatosis in alcohol-fed mice (183). Indeed, miR-132 is elevated in the liver of AUD patients with fibrosis/cirrhosis (180). Other alcohol-induced miRNAs contribute to dysregulation of monocyte/macrophage cytokine responses including miR-217 which also regulates TGF-β expression in Kupffer cells of alcohol-fed mice (184), as well as miR291b and miR-181b-3b both of which are negative regulators of the TLR signaling pathway (185, 186).

In addition to their effect on the production of cytokines and chemokines, alcohol-induced miRNAs affect other monocyte/macrophage cell functions including oxidative stress and phagocytosis. Chronic drinking downregulates miR-92a and upregulates miR-130a and miR-301a in mouse alveolar macrophages leading to increased oxidative stress and reduced bacterial phagocytosis (25, 187). Downregulation of miR-92a leads to upregulation of Nox4 (25) while upregulation of miR-130a and miR-301a reduces PPARγ expression leading to increased gene expression of Noxes 1, 2, and 4 (187). Nox upregulation results in increased oxidative stress response and increased production of TGF-β as well as decreased phagocytosis (25, 187, 188). ROS production by Kupffer cells is also upregulated by alcohol-induced miR-217 (184).

Alcohol-induced changes in miRNA expression can modulate macrophage polarization balance. LPS-stimulated Kupffer cells from alcohol-exposed rats show enrichment of miR-125a-5p (189), which inhibits TLR4-dependent signaling pathway and mediator production leading to anti-inflammatory polarization (189). Moreover, miR-27a, which regulates macrophage polarization towards a regulatory profile *via* IL-10, is downregulated in PBMCs of ethanol-fed NHPs (21) suggesting an alcohol-induced rewiring towards a pro-inflammatory profile.

In addition to regulating gene expression within the cells in which they are generated, miRNAs can be transferred into other target cells *via* exosomes, thus regulating the function of other cell populations including monocytes and macrophages in a paracrine manner (174, 190, 191). Of note, increased miR-122 levels have been measured in circulating exosomes of chronic alcohol-fed mice (140) and binge-drinking humans (191). miR-122 is liver-specific and is associated with lipid metabolism, stress response, and hepatitis C virus replication (192). *In vitro*, horizontal transfer of miRNA-122 from ethanol-treated hepatocytes to monocytic cells *via* exosomes upregulates the production of pro-inflammatory cytokines TNF-α and IL-1β (191). In PBMCs isolated from heavy drinking NHPs, alcohol exposure had a differential effect on several extracellular vesicle (EV)-derived miRNAs that regulate genes with roles in myeloid cell activation and angiogenesis. Gene ontology analyses revealed enrichment of putative target genes to TGF-β receptor signaling, histone, and chromatin modification, response to ROS, and myeloid leukocyte activation. Over-expression of candidates miR-155, miR-154, miR-34c, miR-450a, and miR-204, which are upregulated in EV with alcohol drinking, led to a heightened inflammatory response in PBMCs after stimulation (190).

### DNA Methylation

DNA methylation plays an important role in ethanol-mediated dysfunction. In Kupffer cells, alcohol treatment leads to abnormal DNA methylation patterns and upregulation of methyl-transferases DNMT1, DNMT3a, and DNMT3b (193) which in turn hyper-methylate anti-inflammatory mediators, *PSTPIP2*, *SOCS1*, and *ZSWIM3* leading to their inhibition and inhibition of downstream anti-inflammatory targets and promotion of a pro-inflammatory profile (193). Specifically, downregulation of *PSTPIP2* leads to activation of the STAT1 pathway *via* increased STAT1 phosphorylation. Downregulation of *PSTPIP2* also leads to activation of the NF-κB signaling pathway by affecting phosphorylation of p65 and IκBα as well as the nuclear transfer of p65. Activation of these pathways results in increased transcription of pro-inflammatory mediators IL-1β, TNF-α, IL-6, IL-17, and CCL2 and increased protein production of IL-1β, TNF-α, and IL-6. Thus, *PSTPIP2* plays a crucial role in macrophage-induced inflammatory responses by regulating the STAT1 and NF-κB signaling pathways (193). In the same chronic-plus-binge mouse model, alcohol exposure also reduces the expression of *ZSWIM3* in Kupffer cells due to DNMT3b-induced hypermethylation of *ZSWIM3* promoter. Downregulation of *ZSWIM3* leads to activation of adaptor protein TRAF2 which plays a crucial role in the activation of the NF-κB signaling pathway resulting in increased transcription of IL-1β, TNF-α, IL-6 and MCP-1 as well as increased protein production of IL-1β, TNF-α and IL-6 (194) thus eliciting a pro-inflammatory phenotype.

### Histone Modifications

Chronic alcohol exposure leads to histone modifications that contribute to enhanced pro-inflammatory responses. Sirtuins are NAD+ dependent deacetylases that play a key role in inflammation and oxidative stress. Ethanol metabolism results in a decrease of NAD+ in the liver leading to sirtuin dysregulation. Additionally, alcohol consumption leads to the upregulation of miR-217 and miR-132 in Kupffer cells, which in turn leads to decreased levels of sirtuin SIRT1 (184, 195). Decreased SIRT1 gene and protein expression results in the downregulation of AMPK, an anti-inflammatory regulator, and the upregulation of NF-κB and NFATC4 through H3K9 acetylation. Activation of the NF-κB and NFATC4 signaling pathways results in increased production of pro-inflammatory IL-1β, TNF-α, IL-6 and MCP-1 (184). In addition to sirtuins, other classes of deacetylases have been involved in alcohol-induced effects. Histone deacetylase 11 (HDAC11) is induced in Kupffer cells in a mouse model of ALD *via* upregulation of miR-155 by alcohol metabolite acetaldehyde that leads to activation of the NF-κB signaling pathway, resulting in reduced expression of anti-inflammatory IL-10 (179). The mechanism of action of HDAC11 in macrophages has been elucidated. HDAC11 binds to the proximal site of the IL-10 promoter and modulates the recruitment of PU.1, Sp1, and STAT3 at late stages of LPS activation (196).

Histone methylation is also affected by alcohol consumption. H3K27me3, mediating epigenetic silencing, is enriched at the TGF-β promoter but reduced at the TNF-α promoter location in Kupffer cells of ACLF patients. Enhancer of zeste homolog 2 (EZH2) catalyzes the methylation of H3K27 loci and increased EZH2 expression and enhanced H3K27me3 are observed in PBMCs of ACLF patients. In addition, in a murine model of liver failure induced by administration of D-galactosamine, upregulation of EZH2 and H3K27me3 in Kupffer cells correlates with upregulation of pro-inflammatory cytokine TNF-α, IL-1β, and IL-6 (197). Furthermore, in splenic macrophages of alcohol-fed NHPs, chronic alcohol affects increased tri-methylation of H3K4, an active promoter mark. In addition, changes in overlapping regions enriched for cis-regulatory inactive enhancer H3K4me1 and active enhancer H3K27Ac are associated with host defense processes including “Immune System Process” and “regulation of cytokine production” in alcohol-exposed splenic macrophages (22).

### Chromatin Accessibility

Changes in histone modifications can lead to alterations in chromatin accessibility, resulting in changes in gene expression. Several studies have reported modulation of chromatin accessibility in monocytes and macrophages from alcoholic hepatitis (AH) patients and NHP chronically consuming ethanol leading to enhanced pro-inflammatory gene expression and reduced expression of genes involved in metabolic processes (22, 105, 118). In alveolar macrophages of alcohol-fed NHPs, these dysregulated responses are mediated by increased chromatin accessibility in intergenic regions that regulate inflammatory genes and binding motifs for transcription factors AP-1, IRF8, and NF-κB p-65 and reduced chromatin accessibility of promoters of genes important for endothelium development and cell junction assembly (105). In splenic macrophages of alcohol-fed NHPs, chronic alcohol increases chromatin accessibility of promoters and intergenic enhancer regions that regulate the expression of genes participating in immune activation, cytokine signaling pathways, myeloid cell activation (CD40), and cellular stress responses. Specifically, chronic alcohol increases accessibility in regions regulated by transcription factors involved in oxidative stress (NRF-1, AHR), hypoxia (HIF-1α), and inflammatory responses (NF-κB). Chronic alcohol is also associated with increased chromatin accessibility of genes engaged in inflammatory and immune responses including *TLR4*, *CCL2*, *C3AR1*, and *LAMP1* (22).

In addition to its effect on tissue macrophages, chronic heavy alcohol consumption also increases chromatin accessibility at promoter regions that regulate the expression of genes involved in cytokine production and myeloid activation in monocytes of ethanol-fed NHPs, including regions containing binding sites for transcription factors important for monocyte activation and differentiation such as FOS, JUNB and PU.1 (68). Furthermore, studies reported increased accessibility to binding sites for transcription factors CEBP and MAF as well as regions associated with immunoregulatory genes such as *PPARG* in monocytes isolated from patients with severe AH. In contrast to what has been described for monocytes of heavy drinkers in the absence of liver disease, binding sites for NF-κB, IRF, and STAT1 were less accessible in enhancer regions as was chromatin accessibility in regions associated with genes involved in innate immune response, antigen presentation and cytokine secretion in monocytes obtained from patients with AH (118).

## Impact of Aud on Bone Marrow Myeloid Progenitors

Monocytes develop continuously in the bone marrow from CD34+ hematopoietic stem and progenitor cells *via* increasingly restricted lineage-committed progenitors (10, 11). Inflammation and infection can affect monocyte production and induce emergency monopoiesis in the bone marrow (10, 198). AUD patients suffer from blood cell disorders including lymphopenia, anemia, and thrombocytopenia (199–203). In addition, almost half of alcoholics have foamy macrophages due to the storage of alcohol-induced lipids in the cytoplasm (204). Hemostasis is also perturbed in ALD, due to alcohol’s effects on the platelets leading to an increased risk of disseminated intravascular coagulation (205). These defects suggest that alcohol exposure impacts bone marrow progenitor cell populations and hematopoiesis in humans. Alcohol-induced effects on the myeloid progenitor compartment are wide-ranging, impacting hematopoietic precursor cell activation and differentiation as well as genes involved in inflammatory and metabolic responses. Multiple bone marrow precursor cell populations are affected by chronic alcohol, including erythrocytes and granulocytes where vacuolization is induced. This damage appears within a week of heavy drinking but is reversible (205).

Mice exposed to acute alcohol and infected with *E.coli* have a reduced number of bone marrow myeloid progenitor cells and alcohol treatment prevents switching to granulocyte phenotype. Furthermore, alcohol exposure increases the bacterial burden and mortality of E.coli-infected mice (206). In a chronic plus binge mouse model, alcohol administration triggers the release of granulocytes from the bone marrow compartment, resulting in a reduction of the granulocyte reserve in the marrow concomitant with an elevation of granulocytes in the circulation. This phenomenon is enhanced further following bacteremia, where alcohol impairs activation of granulopoietic precursor proliferation and response to LPS stimulation (207). These findings were confirmed in NHPs, where chronic alcohol exposure reduces the number of primitive hematopoietic stem and progenitor cells in the bone marrow and impairs their ability to proliferate and differentiate towards erythroid and granulocyte-monocyte cells (208). In addition, alcohol exposure leads to remodeling of the bone marrow niche and these alterations persisted after 1-month abstinence thus indicating that the effects of alcohol are long-lived (208) and may involve epigenetic rewiring of progenitor cells.

## Conclusion

In summary, chronic alcohol consumption leads to a hyper-inflammatory response with heightened cytokine, chemokine, and ROS production concomitantly with decreased microbial and wound healing responses in circulating monocytes and tissue-resident macrophages. This profile is due to alcohol’s interference with the fundamental processes of epigenetic regulation at the transcriptome level.

## Author Contributions

All authors listed have made a substantial, direct, and intellectual contribution to the work and approved it for publication. IM secured funding for this work.

## Funding

This work was funded by NIH Grant number R01 AA028735.

## Conflict of Interest

The authors declare that the research was conducted in the absence of any commercial or financial relationships that could be construed as a potential conflict of interest.

## Publisher’s Note

All claims expressed in this article are solely those of the authors and do not necessarily represent those of their affiliated organizations, or those of the publisher, the editors and the reviewers. Any product that may be evaluated in this article, or claim that may be made by its manufacturer, is not guaranteed or endorsed by the publisher.
